# FibroScan^®^ versus Biochemical Scores: A Study of Liver Fibrosis in HIV with HBV Co-Infection

**DOI:** 10.3390/microorganisms12061213

**Published:** 2024-06-16

**Authors:** Giorgiana Nicoleta Lungu, Gheorghe Iulian Diaconescu, Florentina Dumitrescu, Anca Oana Docea, Radu Mitrut, Lucian Giubelan, Ovidiu Zlatian, Paul Mitrut

**Affiliations:** 1 Doctoral School, University of Medicine and Pharmacy of Craiova, 200349 Craiova, Romania; lungugiorgiananicoleta@gmail.com (G.N.L.); radumitrut@yahoo.co.uk (R.M.); 2 “Victor Babes” Infectious Diseases and Pneumophtisiology Clinical Hospital, 200515 Craiova, Romania; diaconescu_ig@yahoo.com (G.I.D.); dumitrescu_florentina@yahoo.com (F.D.); ligiubelan@yahoo.com (L.G.); 3 Department of Infectious diseases, University of Medicine and Pharmacy of Craiova, 200349 Craiova, Romania; 4 Department of Toxicology, University of Medicine and Pharmacy of Craiova, 200349 Craiova, Romania; ancadocea@gmail.com; 5 Microbiology Department, University of Medicine and Pharmacy of Craiova, 200349 Craiova, Romania; 6 Medical Laboratory, County Clinical Emergency Hospital of Craiova, 200349 Craiova, Romania; 7 Department of Internal Medicine, University of Medicine and Pharmacy of Craiova, 200349 Craiova, Romania; paulmitrut@yahoo.com; 8 Department of Internal Medicine II, County Clinical Emergency Hospital of Craiova, 200642 Craiova, Romania

**Keywords:** HIV, hepatitis, HBV, fibrosis, transient elastography, FIB-4, APRI

## Abstract

The study aimed to determine liver fibrosis in human immunodeficiency virus (HIV) positive individuals using transient elastography (FibroScan^®^), Fibrosis-4 (FIB-4) score, and aspartate aminotransferase (AST) to Platelet Ratio Index (APRI) in the HIV Department from Infectious Diseases Hospital “Victor Babeș” Craiova, Romania. Of the analyzed HIV-positive subjects (*n* = 161), 93 (57.76%) had HIV mono-infection, and 68 (42.24%) had Hepatitis B Virus (HBV) co-infection. The prevalence of advanced liver fibrosis was higher (F2: 11.76% and F3: 13.24%, F4: 4.41%) in the HIV-HBV co-infected group compared to the HIV mono-infected group. The univariate and multivariate analysis identified HBV co-infection (OR = 5.73) male sex (OR = 5.34), serum aspartate amino-transferase levels (Pearson’s rho = 0.273), low platelet count (Pearson’s rho = −0.149) and erythrocyte sedimentation rate (OR = 1.030) as risk factors for the presence of liver fibrosis. Body mass index (OR = 1.08), serum lipid levels (OR = 0.96), viral load at diagnosis (OR = 1.00005), and low CD4+ cell count (OR = 0.977) were also correlated with liver fibrosis. The FIB-4 and APRI scores were strongly correlated with each other. In conclusion, HBV co-infection seems to be a determinant factor for liver fibrosis development in people living with HIV, together with other risk factors.

## 1. Introduction

Human immunodeficiency virus-1 (HIV) infection is a major global public health issue. By the end of 2022, it was estimated that 39.0 million (33.1–45.7 million) people are living with HIV infection worldwide, two-thirds of whom live in the WHO Africa Region [1].

According to the latest estimates, there were 296 million people worldwide suffering from chronic Hepatitis B Virus (HBV) infection in 2019 and an additional 1.5 million new infections each year [2]. The precise number of individuals with HIV-HBV co-infections is unknown, but a recent review of global data suggests that the prevalence of HIV-HBV co-infections is 7.6% (IQR 5.6–12.1), or approximately 2.7 million people co-infected. This indicates that 1 out of every 100 individuals with HBV infection also has HIV infection [3].

In Romania, 18,015 people living with HIV were reported by the end of June 2023 [4]. It was established as a target for 2025, which indicates that 95% of people living with HIV(PLHIV) should have a diagnosis, 95% should receive antiretroviral therapy (ART), and 95% of PLHIV who are receiving ART should achieve undetectable viral loads to reduce HIV transmission and for human health and survival [1].

Since 1996, when highly active antiretroviral therapy (HAART) was introduced, the human life expectancy of PLHIV has increased over the past 25 years and has become similar to that of the general population if treatment is started at high CD4 cell counts [5]. The frequency of deaths due to acquired immunodeficiency syndrome(AIDS)-defining illnesses has decreased in the era of modern ART. However, deaths due to non-AIDS-defining illnesses such as liver disease, cardiovascular events, and non-AIDS malignancies have been on the rise [6]. Therefore, to achieve this goal by 2025, it is necessary to diagnose and control non-AIDS-defining illnesses. In PLHIV, liver-related diseases vary between 13% and 18% [7] of all causes of mortality; therefore, it is one of the main causes of death unrelated to acquired immunodeficiency syndrome [7]. Unlike the general population, people living with HIV, even if the infection is under control, are more likely to develop complications due to liver disease because of common causes, such as alcoholism, fatty liver disease, hepatic viral infections, and aging, to which other specific causes are added, related to HIV infection. HIV by itself and glycoprotein 120 can induce the activation of hepatic stellate cells (these cells are the most important involved in fibrogenesis and the source for extracellular matrix (ECM) [8] that promotes inflammation and fibrosis [9]) and drug-induced liver injury due to antiretroviral therapy [10]. There are various studies that sustain the effect of HIV itself on liver inflammation and progression to fibrosis: the liver is responsible for the clearance of viral particles from the blood as demonstrated in studies conducted on mice and rhesus monkeys infected with HIV [11,12], which promotes inflammation and cytokine release; HIV is binding with coreceptors like C-X-C chemokine receptor type 4 (CXCR-4) and C-C chemokine receptor type 5 (CCR-5), which are expressed on hepatocytes and promote the production of alpha-1 procollagen [13]; there is an increased rate of apoptosis of hepatocytes due to intracellular replication of HIV [14] that promotes inflammation and consequently the evolution to fibrosis; hepatocytes are raising the expression of transforming growth factor beta-1 (TGF β-1) like a response to the presence of HIV [15]. These are some of the mechanisms through which HIV can cause liver damage and evolution towards liver fibrosis, which is why people living with HIV should be carefully monitored and investigated regarding the risk of developing liver fibrosis and should be investigated to identify liver fibrosis.

In various models of HBV infection, it has been noted that liver injuries in chronic infection are linked to the activity of HBV-specific T cells [16]. Studies have shown that molecular and cellular changes in host gene expression are supported by virus replication, which protects virally infected cells from immune-mediated destruction and promotes tumorigenesis. The inflammation-induced oxidative stress activates Kupffer cells, which in turn stimulate stellate cells through NF-κB and AP1 pathways [16]. Non-invasive methods, such as transient elastography and serum biomarkers, are used to assess the extent of fibrosis (scarring) in organs, particularly the liver, without the need for a biopsy. These tests are essential for diagnosing and monitoring liver diseases like hepatitis, fatty liver disease, and cirrhosis. The benefits of these non-invasive methods include avoiding the risks and discomfort associated with liver biopsy, repetition, as they can be performed multiple times to monitor disease progression or response to treatment, and convenience, as they allow for outpatient settings without the need for hospital admission [17].

Using TE for staging liver fibrosis is recommended by the latest guidelines from the European Association for the Study of the Liver (EASL, 2021) [18], the World Health Organization (WHO, 2022) [19], the American Association for the Study of Liver Diseases (AASLD, 2018) [20], and the British HIV Association [21] endorse the use of non-invasive methods, such as transient elastography (FibroScan^®^), to assess liver fibrosis and recommend specific breakpoints for TE that we used. These guidelines highlight the importance of liver fibrosis staging in managing chronic liver diseases, particularly in patients with HBV and HIV co-infection, which are complex patients who are influenced by a multitude of factors that affect the progression of hepatic fibrosis. Additionally, these patients are immunocompromised, making them more susceptible to opportunistic infections or neoplasms, and the treatments and progression of these conditions can lead to a much faster evolution of hepatic fibrosis.

Present evidence from past research [22] where liver fibrosis stages (F0–F4) were effectively used to assess liver disease progression and outcomes in HIV and HIV-HBV co-infected individuals.

This study aimed to determine the presence of liver fibrosis in people living with HIV with the help of non-invasive methods such as transient elastography(FibroScan^®^), liver fibrosis scores like Fibrosis-4 (FIB-4) and AST to Platelet Ratio Index(APRI) and identify the potential risk factors associated with the development of liver fibrosis in a cohort of people living with HIV regularly attending the HIV Department from Infectious Diseases Hospital “Victor Babeș” Craiova, Romania.

## 2. Materials and Methods

### 2.1. Patients

The present study represented a cross-sectional prospective study that included 161 patients living with HIV who attended the HIV Department, which is part of the Infectious Diseases Hospital “Victor Babeș” Craiova, Romania, between January 2022 and June 2023.

In the current records from the HIV Department at the Infectious Diseases Hospital “Victor Babeș” in Craiova, Dolj county, there are 423 patients, as reported by the National Department for Monitoring and Evaluating HIV/AIDS infection in Romania [4].

The patients included in this study were those who had either HIV mono-infection or HIV plus HBV co-infection. To ensure the results were as relevant as possible, we excluded patients with additional risk factors that could cause liver damage, which could potentially lead to liver fibrosis. Therefore, the exclusion criteria were chronic alcohol consumption, patients undergoing antituberculosis, antifungal, or chemotherapy treatment at the time of the study, as well as those with other co-infections, such as hepatitis C or D, Cytomegalovirus infection, or active tuberculosis.

After excluding these patients, only 161 patients were included in this study. The patients were evaluated as they presented to the HIV Department at the hospital for their monthly clinical evaluation and for lifting antiretroviral therapy (in Romania, ART is released from the hospital pharmacy monthly only to the patient or the legal representative).

Of the 161 patients, 93 (57.76%) were HIV positive, and the remaining 68 (42.24%) had HBsAg positive seropositivity (HBV infection) for more than six months, therefore representing the HIV plus HBV co-infection group. HIV infection was diagnosed using serological enzyme-linked immunosorbent assay (ELISA), and positive ELISA tests were confirmed by Western Blot analysis tests.

All patients signed an informed consent stating the objectives and the benefits of the study. The study design was approved by the Human Research Committee of the University of Medicine and Pharmacy, Craiova, and it conformed to the ethical guidelines of the Declaration of Helsinki in 1975. The study protocol followed the ethical guidelines of the Declaration of Helsinki and was approved by the Ethics Committee of the University of Medicine and Pharmacy of Craiova (Approval No. 78/07.09.2020).

### 2.2. Liver Fibrosis Measurement

Liver stiffness measurement was performed once for patients with normal measurement and twice by two professional workers for those with abnormal liver stiffness measurement. FibroScan^®^ was performed only on patients who had a minimum 3 h fast since their last meal.

Using transient elastography (FibroScan^®^, ECHOSENS, Paris, France), we assessed liver stiffness with a FibroScan^®^ 502 Touch (ECHOSENS, Paris, France). Measurements were performed by trained operators with experience who were aware of the patient’s pathology and clinical context. Liver stiffness was expressed in kilopascals (kPa), and the result is the median of all valid measurements performed during the examination (following the instruction manual; to obtain a reliable and representative liver stiffness measurement, at least 10 valid measurements were performed). For the examination to be considered reliable, the interquartile range (IQR) over the median (IQR/MED) percentage should not exceed 30%.

Blood samples were collected for biochemical analysis on the same day as the FibroScan^®^ was performed. Based on these results, the current APRI and FIB-4 scores were calculated.

When patients were diagnosed with HIV infection or HIV plus HBV co-infection, blood tests were performed. The baseline (initial) APRI and FIB-4 scores were calculated based on the test results.

Liver stiffness was assessed using non-invasive methods such as transient elastography (FibroScan^®^), FIB-4 score (Fibrosis-4), and AST to platelet ratio index (APRI). Because antiretroviral therapy can influence transaminase levels and implicit FIB-4 and APRI scores, we used transient elastography to improve the accuracy of the measurement of liver fibrosis.

FIB-4(Fibrosis-4) score. We calculated the FIB-4 score according to the formula for FIB-4 as (age [years] × AST [U/L])/(platelet count [10^9^/L]) × sqrt(ALT [U/L])), as recommended by Sterling et al. [23].

APRI-score. The APRI score was calculated [24] using the following formula: APRI = (AST Level (IU/L)/AST (Upper Limit of Normal) (AST))/(platelet count (10^9^/L)) × 100.

The breakpoints for the presence of liver fibrosis for the three fibrosis scores are defined as follows:

- FibroScan^®^: F0/F1 fibrosis: lower than 7.4 kPa; F2 fibrosis: 7.4–9.5 kPa; F3 fibrosis: 9.6–12.5 kPa; F4 fibrosis: equal or greater than 12.6 kPa;
- FIB-4 score: the presence of fibrosis: higher than 1.45;
- APRI score: the presence of fibrosis: greater than 0.5.

As part of the laboratory examination, the following parameters were determined: glutamic pyruvic transaminase (ALT), glutamic oxaloacetic transaminase (AST), serum urea, creatinine (CRE), whole blood count, HIV viral load, and CD4 and CD8 lymphocyte counts. The glomerular filtration rate (EGFR) was calculated [11] as EGFR = ((140-age) × weight (kg))/(7.2 × seric creatine).

Biochemical markers were measured using an Architect C8000 analyzer (Abbott Corporation, Green Oaks, IL, USA), which employed closed-system reagents produced by the same company. The levels of the liver enzymes (TGO and TGP) were determined using an enzymatic method. CRE and total lipid contents were determined using an enzymatic colorimetric method. All patients provided written consent and were informed of the procedures involved in the study as well as their right to withdraw at any time once included. Whole blood count was determined using a Cell Dyn Ruby (Abbott Laboratories, USA) hematology analyzer. CD4 and CD8 T lymphocyte counts were determined using a standard flow cytometer, and the viral load was determined using a Cobas (Roche Diagnostics, Basel, Switzerland) genetic analyzer.

### 2.3. Statistical Analysis

The acquired data were saved in Microsoft Excel files (Microsoft Corp., Redmond, WA, USA), and statistical analysis was conducted using STATA 17.0 SE (StataCorp LLC, College Station, TX, USA). Data were presented as the median and interquartile range (IQR), defined as the interval between the 25th and 75th percentile. Student’s *t*-test was used to compare groups with a normal distribution of values, while the Wilcoxon signed-rank test was used for groups with a non-normal distribution. Dunn’s post hoc multiple comparison test was used to assess differences between the groups. STATA’s multiple logistic regression analysis with the robust standard errors option was employed for regression analysis, ROC curves, and method classification performance. Graphical representations were created using STATA 17.0 SE software. Statistical tests for differences between groups were considered significant when the significance level (*p*-value) was <0.05.

## 3. Results

### 3.1. Descriptive Statistics

We compared clinical and paraclinical parameters between patients living with HIV and patients co-infected with HIV+HBV (Table 1).

Patient demographics and mode of transmission. Patients with HIV had a median age of 35 years, with sexual contact being the primary mode of transmission (70.65%). In contrast, patients co-infected with HIV and HBV had a median age of 33 years, with parenteral transmission being the predominant mode (70.59%). The transmission routes differed significantly between the two groups of patients. The median age at diagnosis was significantly lower (11.5 years) in HIV+HBV co-infected patients than in people living with HIV.

Clinical parameters and treatment duration. People living with HIV had a median antiretroviral treatment duration of 11 years, predominantly falling under stage C3 (36.56%). Co-infected patients had been on treatment for 22.5 years, significantly higher than people living with HIV, with a similar stage distribution.

Immunological status. At diagnosis, HIV patients had a median CD4 count of 251 cells/mm^3^, increasing to 401 cells/mm^3^ over time. Co-infected patients had slightly higher initial (266.5 cells/mm^3^) and current (445 cells/mm^3^) CD4 counts. Both groups had comparable CD8 counts and CD4/CD8 ratios.

Virological status. Initial HIV viral load was significantly higher in the HIV-only group (26,400 copies/mL) compared to the co-infected group (7517.5 copies/mL). At the study’s time, a higher percentage of co-infected patients had suppressed viral loads (75.76% vs. 67.39%).

Liver function tests. Median AST and ALT levels were similar but slightly elevated in co-infected patients (both at 33 U/L) compared to HIV-only patients (AST: 28 U/L, ALT: 27 U/L).

Renal function and other metabolic parameters. Renal function was similar in both groups, with creatinine levels and eGFR values being nearly identical. BMI was slightly lower in co-infected patients (23.66) compared to HIV-only patients (23.81).

Liver fibrosis assessment. Liver fibrosis in people living with HIV, measured using FibroScan^®^, showed that at the time of the study, most patients had mild fibrosis in stages F0–F1 (91.4%). Only a minority were in more advanced stages (F2: 5.38%, n = 5; F3: 1.08%, n = 1; F4: 2.15%, n = 2). Initial and current FIB-4 scores were 0.69 (IQR: 0.36–1.14) and 0.9 (IQR: 0.68–1.54) respectively. APRI scores at diagnosis and at the time of the study were 0.36 (IQR: 0.22–0.83) and 0.34 (IQR: 0.23–0.57), both suggesting low levels of liver fibrosis in the cohort. The variability of the fibrosis scores was high, as assessed using the chi-square test (*p* < 0.001).

In patients co-infected with HIV and HBV, the FibroScan^®^ test also showed predominantly mild liver fibrosis at stages F0–F1 (70.59%); however, a significant number of patients also had stages F2 (11.76%) and F3 (13.24%). The FIB-4 scores were lower at diagnosis (0.38, IQR: 0.16–0.77) but showed an increase in the current measurements (0.845, IQR: 0.65–1.34). APRI scores were relatively stable: 0.42 (IQR: 0.225–0.74) at diagnosis to 0.36 (IQR: 0.23–0.53) at the time of the study.

The co-infected cohort was characterized by a longer duration of antiretroviral treatment, a higher proportion of parenteral transmission, and slightly better immunological markers than the mono-infected group. Although liver function tests and fibrosis indicators suggested a marginally higher burden of liver disease, the majority of patients in both groups had well-controlled HIV infection and largely normal liver and renal functions. However, the co-infected group showed a higher prevalence of advanced fibrosis stages based on FibroScan^®^ results.

We can conclude that the co-infected cohort showed significant differences in the modes of HIV transmission, distribution across the clinical and immunological HIV stages, rates of viral suppression, and stages of liver fibrosis. However, there were no significant differences in the sex or body weight status distributions.

### 3.2. Comparison of Parameter Distributions between the Two Patient Groups

The sex distribution did not differ significantly between the two groups (*p* = 0.645). Additionally, the median age differed between the two groups (*p* < 0.001), as did treatment duration (*p* < 0.001). A significant difference was observed between the modes of transmission (*p* < 0.001). The clinical and immunological stages of HIV infection showed no significant differences in distribution between the two groups (*p* = 0.814). The median ESR was not significantly different between the two groups (20 vs. 22.5 mm/1 h, *p* = 0.734). Analysis of the proportion of patients with a viral load of <40 c/mL revealed no significant difference between the groups (*p* = 0.335). The proportion of overweight patients did not differ significantly between the two groups (*p* = 0.918).

The staging of liver fibrosis assessed using FibroScan^®^ differed between the two groups, with an increased proportion of patients in stages F2 and F3 (*p* = 0.003). The median FIB-4 score at the time of HIV diagnosis was almost halved (0.38 vs. 0.69) in the co-infected group.

Significant differences were observed between the two groups in terms of the modes of transmission and stages of liver fibrosis. The co-infected group also differed significantly in terms of age and treatment duration. No significant differences were observed in sex distribution, AIDS stage, viral suppression rate, or body weight.

**Figure 1:**
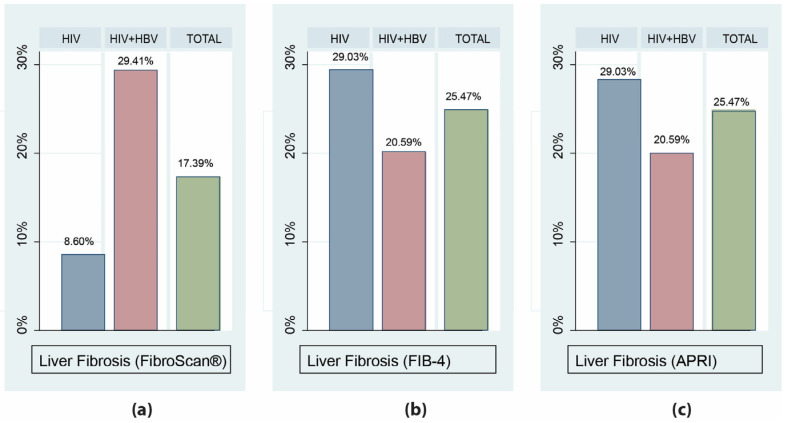
Proportions of patients with significant liver fibrosis among patients living with HIV alone or co-infected with HBV, as assessed by FibroScan^®^ (**a**), FIB-4 (**b**), and APRI scores (**c**).

**Figure 2:**
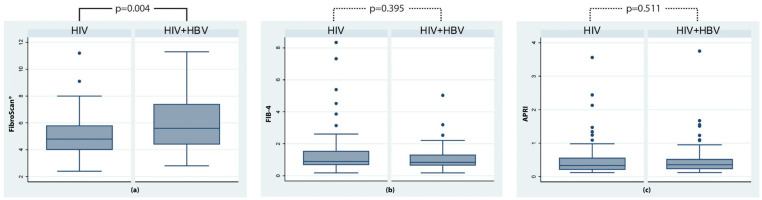
Differences in fibrosis scores between the two groups, as assessed by FibroScan^®^ (measured in kilopascals, kPa) (**a**), FIB-4 (results expressed in points) (**b**) and APRI (results expressed in points) (**c**) scores.

Liver fibrosis was staged according to the parameters measured as non-significant and present. In the group of patients living with HIV, only 8.60% presented with liver fibrosis, as assessed using transient elastography (FibroScan^®^), whereas in the group of patients co-infected with HIV and HBV, 29.41% presented with liver fibrosis (Figure 1). These findings were confirmed by the analysis of raw FibroScan^®^ scores (Figure 2), which demonstrated a significantly higher median score in the co-infected groups (*p* = 0.004). There were no differences in the percentage of patients classified as having fibrosis, as assessed by the fibrosis scores of FIB-4 and APRI (29.03% in the first group and 20.59% in the second group) (Figure 1).

### 3.3. Association between Fibrosis Scores and Potential Risk Factors

By analyzing the sex distribution of fibrosis scores, we observed higher scores in men, which reached statistical significance for FIB-4 (*p* = 0.004) and APRI (*p* = 0.005) scores in the patients living with HIV and FibroScan^®^ groups (*p* = 0.025), and APRI (*p* = 0.021) scores in the co-infected group.

**Figure 3:**
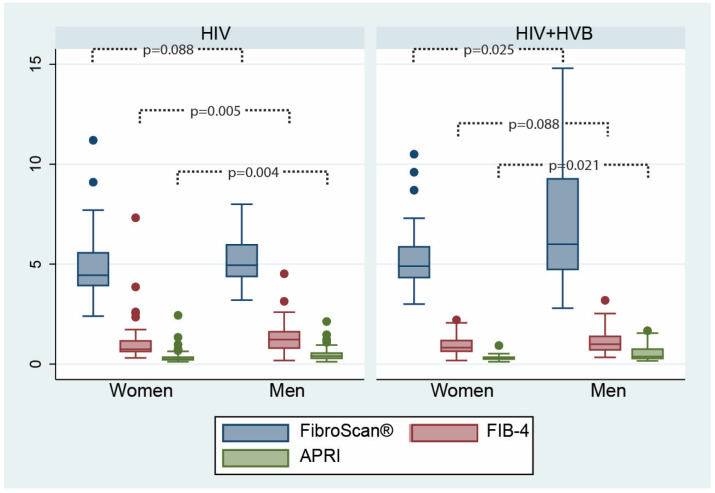
Differences in fibrosis scores according to sex between the two patient groups: FibroScan^®^ (measured in kilopascals, kPa), FIB-4 (results expressed in points), and APRI (results expressed in points) scores.

For patients living with HIV, the FIB-4 (*p* = 0.005) and APRI (*p* = 0.004) scores showed significant sex differences (Figure 3), whereas in the co-infected group, both the FibroScan^®^ and APRI scores showed significant differences (*p* = 0.025 and *p* = 0.021, respectively). The results of the sex distribution of liver fibrosis suggest that male patients, particularly those co-infected with HBV, may have a higher degree of liver fibrosis than female patients, although this trend does not apply uniformly across all fibrosis scoring methods.

**Figure 4:**
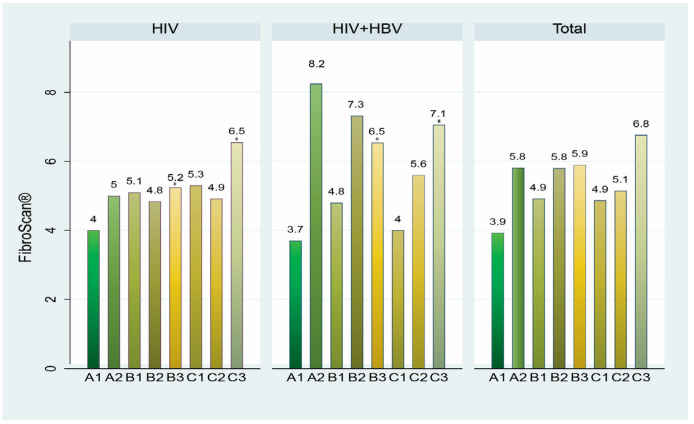
Correlation between clinical and immunological AIDS staging and liver fibrosis, as assessed by transitory elastography (FibroScan^®^) in patients living with HIV and those co-infected with HIV and HBV. * = *p* < 0.05 for the difference between the HIV and HIV+HBV groups.

Transient elastography scores were analyzed according to clinical and immunological AIDS staging (Figure 4). In patients living with HIV, the highest mean score was observed in C3 (6.5) and the lowest in lot A1 (4). In patients co-infected with HIV and HBV, the scores were generally higher, ranging from 3.7 in patients with clinical and immunological HIV infection stages A1 to 8.2 in those with stage A2. There were significant differences between the TE measurements in monoinfected vs. co-infected patients in B3 and C3 clinical and immunological stages (Figure 4).

**Figure 5:**
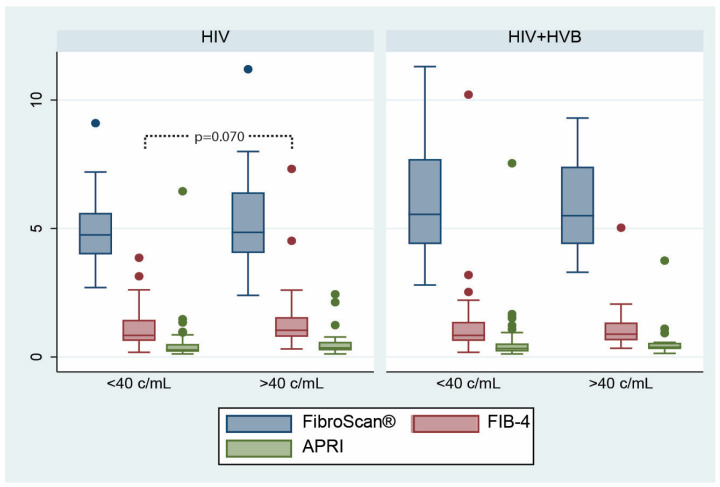
Differences in fibrosis scores according to viral load at diagnosis between the two patient groups: FibroScan^®^ (measured in kilopascals, kPa), FIB-4 (results expressed in points), and APRI (results expressed in points) scores.

None of the fibrosis scores showed a statistically significant difference based on the current viral load for either the HIV-only or HIV+HBV cohorts (Figure 5), although in patients living with HIV, the FIB-4 score was higher in patients with detectable HIV-ARN levels, and this difference approached statistical significance (*p* = 0.07).

**Figure 6:**
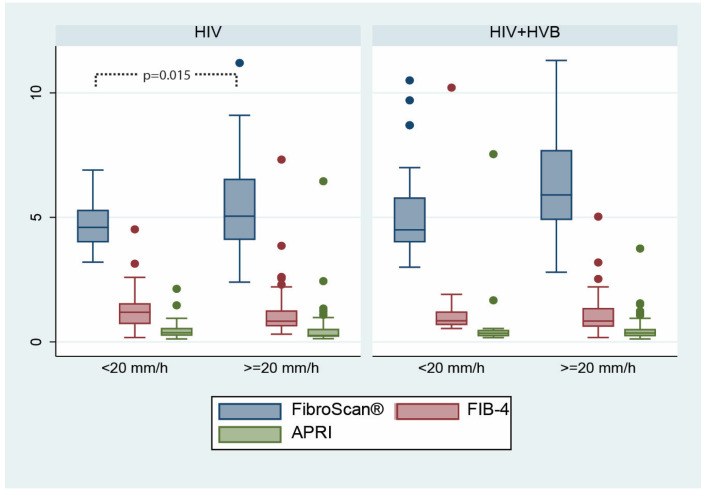
Differences in fibrosis scores according to ESR between the two patient groups: FibroScan^®^ (measured in kilopascals, kPa), FIB-4 (results expressed in points), and APRI (results expressed in points) scores.

Within the HIV mono-infected group, we observed a significant difference in FibroScan^®^ scores between patients with ESR < 20 mm/h and those with ESR ≥ 20 mm/h (Figure 6), suggesting that higher ESR levels may be associated with increased liver fibrosis as measured by FibroScan^®^. Regarding the FIB-4 and APRI scores and all fibrosis measures within the HIV+HBV co-infected group, there were no significant differences based on ESR levels. These findings suggest that, while ESR might be correlated with certain fibrosis markers in patients living with HIV, this correlation does not hold in the presence of HBV co-infection. An alternative explanation is that other fibrosis markers (FIB-4 and APRI) are less sensitive to changes associated with ESR.

**Figure 7:**
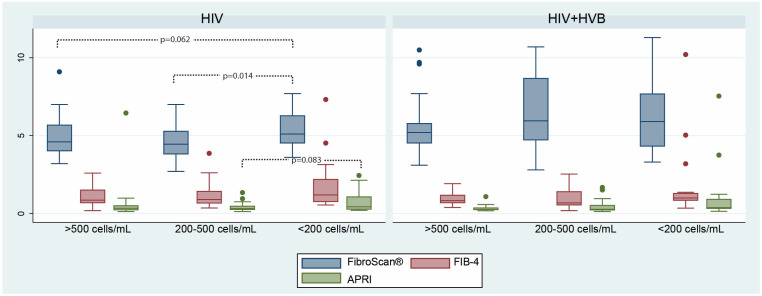
Differences in fibrosis scores according to CD4+ cell count between the two patient groups: FibroScan^®^ (measured in kilopascals, kPa), FIB-4 (results expressed in points), and APRI (results expressed in points) scores.

For the HIV mono-infected group, significant differences in liver fibrosis scores, as measured by FibroScan^®^, were found between the CD4^+^ cell count categories (Figure 7). The Kruskal-Wallis test showed a significant difference among the CD4^+^ cell count categories (*p* = 0.0284). Dunn’s post hoc multiple comparison test revealed significant differences between the <200 cells/mL group and—200–500 cells/mL group (*p* = 0.0138). This suggests that CD4^+^ cell count may correlate with the severity of liver fibrosis in HIV patients without HBV co-infection. For the HIV plus HBV co-infected patients, no significant differences were found across CD4^+^ cell count categories for any of the fibrosis measures, indicating that CD4^+^ cell count levels might not have the same association with liver fibrosis severity in these patients.

### 3.4. Univariate Analysis

Univariate analysis (Table 2) provided insights into the correlation between various parameters and liver fibrosis markers (FibroScan^®^, FIB-4, and APRI) in both patient groups.

Patients living with HIV. Sex did not correlate with liver fibrosis markers, indicating no impact on liver disease progression. Age showed a moderate correlation with FIB-4 (R = 0.230, *p* = 0.0266), implying it may influence liver fibrosis. No significant correlation was observed with BMI, indicating body mass may not affect liver fibrosis. AST levels were strongly correlated with FibroScan^®^ (R = 0.353, *p* < 0.001) and FIB-4 (R = 0.400, *p* < 0.001), making them strong indicators of liver health. Platelet count had a strong negative correlation with FIB-4 and APRI scores (R = −0.582, *p* < 0.001; R = −0.331, *p* = 0.001), linking lower counts with more severe fibrosis. No significant correlations were found with HIV viral load or CD4+ cell count, suggesting these do not directly affect liver health. However, ESR correlated moderately with FibroScan^®^ (R = 0.398, *p* < 0.001), linking inflammation to fibrosis. Time since diagnosis showed a moderate negative correlation with APRI (R = −0.223, *p* = 0.032), suggesting longer time since HIV diagnosis might be associated with less fibrosis.

HIV+HBV co-infected patients. Sex showed a moderate correlation with FibroScan^®^ (R = 0.220, *p* = 0.072), suggesting a potential influence on liver fibrosis. Age at diagnosis was not correlated with fibrosis markers, unlike the HIV-only group, indicating less influence on disease progression. BMI showed a moderate correlation with FibroScan^®^ (R = 0.267, *p* = 0.028), suggesting higher body mass may be linked to more severe fibrosis. Overweight status also correlated moderately with FibroScan^®^ (R = 0.252, *p* = 0.038). AST levels were strongly correlated with FIB-4 and APRI (R = 0.815, *p* < 0.001; R = 0.903, *p* < 0.001), reinforcing their role as key indicators of liver health. ALT levels moderately correlated with APRI (R = 0.239, *p* = 0.050). Platelet count showed strong negative correlations with FIB-4 and APRI (R = −0.520, *p* < 0.001; R = −0.492, *p* < 0.001), similar to the mono-infected group. HIV viral load did not correlate with fibrosis, but CD4+ count showed a moderate correlation with FibroScan^®^ (R = 0.263, *p* = 0.030), indicating that immune status may impact liver health in co-infected patients.

Liver enzymes (AST and ALT) and platelet count (TROMB) were strong indicators of liver fibrosis in both groups. However, there are some interesting differences:

- Sex and age were more relevant in the co-infection group;
- Immune status (CD4+ count) showed some correlation with liver fibrosis in the co-infected group but not in the mono-infected group;
- Body mass index (BMI) and body weight status (overweight) appeared to play a role in liver fibrosis in the co-infected group.

### 3.5. Multivariate Analysis

Multivariate analysis (Table 3) was used to assess the simultaneous contribution to liver fibrosis of several variables, including age, sex, CD4 count, viral load, and treatment duration. Even though some findings contrasted with the univariate analyses, multivariate analysis revealed that HIV-HBV co-infection was independently associated with an increased risk of liver fibrosis.

#### 3.5.1. Correlation between FibroScan^®^ Score and Risk Factors for Liver Fibrosis

The statistical correlation between co-infection with HIV and Hepatitis B (HIV and HBV) and elevated FibroScan^®^ scores (OR = 5.726, *p* = 0.003) suggests a significant increase in the likelihood of advanced liver fibrosis, as indicated in Table 3.

Multivariate analysis indicated a significant association between male sex and a higher FibroScan^®^ score (odds ratio [OR] = 5.338, *p* = 0.014), suggesting that male individuals were at an increased risk of liver fibrosis. In contrast, age at diagnosis was not significantly correlated with liver fibrosis, which was consistent with the findings of the univariate analysis. Therefore, age was not identified as an independent risk factor for liver fibrosis in these cohorts.

The results of the multivariate analysis indicated that BMI was nearly statistically significant (*p* = 0.077), suggesting that body mass may influence liver fibrosis; however, this finding was not conclusive. Additionally, the liver enzymes AST and ALT were not significantly correlated in this model despite their strong univariate correlations with liver fibrosis markers, which was unexpected. Furthermore, platelet count showed no significant correlation, contrary to the univariate findings, suggesting that the univariate correlation may be confounded by other variables.

ESR was significantly correlated with liver fibrosis (OR = 1.030, *p* = 0.005), indicating that higher ESR was associated with more severe liver fibrosis.

Patients living with HIV. Male sex was not significantly associated with liver fibrosis (*p* = 0.182) despite a high OR (11.55). Age at diagnosis was not a factor. Higher serum lipid levels were protective against fibrosis (OR = 0.960, *p* = 0.009), while higher BMI increased the risk (OR = 6.116, *p* = 0.006). AST was positively correlated (OR = 1.739, *p* = 0.006), and ALT negatively correlated with fibrosis (OR = 0.808, *p* = 0.008). Initial HIV viral load and ESR were significant predictors of fibrosis.

HIV+HBV co-infected patients. Male sex showed a strong but not quite significant correlation with liver fibrosis (OR = 7.67, *p* = 0.085). BMI was not statistically significant but suggested a similar trend to the monoinfected group. AST and ALT levels were not correlated, despite expectations. ESR was significantly correlated with fibrosis (OR = 1.028, *p* = 0.045), indicating an increased risk with higher ESR levels.

#### 3.5.2. Correlation between FIB-4 Score and Risk Factors for Liver Fibrosis

Multivariate analysis was used to assess the independent factors affecting the FIB-4 score, a non-invasive marker for liver fibrosis.

Co-infection with HIV and VHB was not significantly associated with FIB-4 fibrosis score. Gender was also not significant, but we noted a rather high odds ratio (OR = 1.991, *p* = 0.124) (Table 4).

Total lipid level was a significant predictor of liver fibrosis (OR = 1.001, *p* = 0.027). Other variables were not statistically significant.

##### Patients Living with HIV 

Male sex was again not statistically significant, but the Odds Ratio was rather high (OR = 2.126, *p* = 0.136), suggesting a potential but inconclusive role of gender. Age at diagnosis was significantly associated with a higher FIB-4 score (OR = 2.94, *p* = 0.041). BMI was nearly significant, with an OR greater than 1 (OR = 1.089, *p* = 0.057), suggesting that a higher BMI could be a risk factor. The total lipid levels were also significant (OR = 1.002, *p* = 0.016).

The other variables were not significant predictors.

##### HIV Plus Hepatitis B Co-Infected Patients

Sex had a high OR (7.197), indicating that men co-infected with HIV and VHB were at a higher risk of developing advanced fibrosis based on the FIB-4 scores. BMI was significantly inversely related to the FIB-4 score (OR = 0.727, *p* = 0.040), in contrast to the mono-infected group.

#### 3.5.3. Correlation between APRI Score and Risk Factors for Liver Fibrosis

Multivariate analysis was used to evaluate the independent factors affecting the APRI score, another non-invasive marker for liver fibrosis.

ALT was positively correlated with the APRI score in both groups, but this could be due to its relationship with AST, which was used to calculate the APRI score. Total lipids were significantly associated with APRI scores only in patients living with HIV patients (OR = 1.003, *p* = 0.039) (Table 5), suggesting a role of lipid profile in the development of liver fibrosis.

### 3.6. Correlations between Liver Fibrosis Assessing Methods

After adjusting for laboratory variables, we calculated the sensitivity and specificity of the FIB-4 and APRI scores using the transient elastography (FibroScan^®^) method and corresponding ROC curves.

FIB-4 and APRI had stronger correlations with each other than FibroScan^®^ in both the groups. The APRI score showed a significant correlation with the FibroScan^®^ only for the entire patient dataset (rho = 0.2079, *p* = 0.008) (Table 6). The high correlation between APRI and FIB-4 in both groups (rho = 0.7905 and 0.7595, respectively) and the total patient dataset (rho = 0.7696, *p* < 0.001) indicated their interchangeability to a certain extent. The correlations between the two scores and TE measurements did not differ significantly between the people living with HIV and patients co-infected with HIV+HBV.

**Figure 8:**
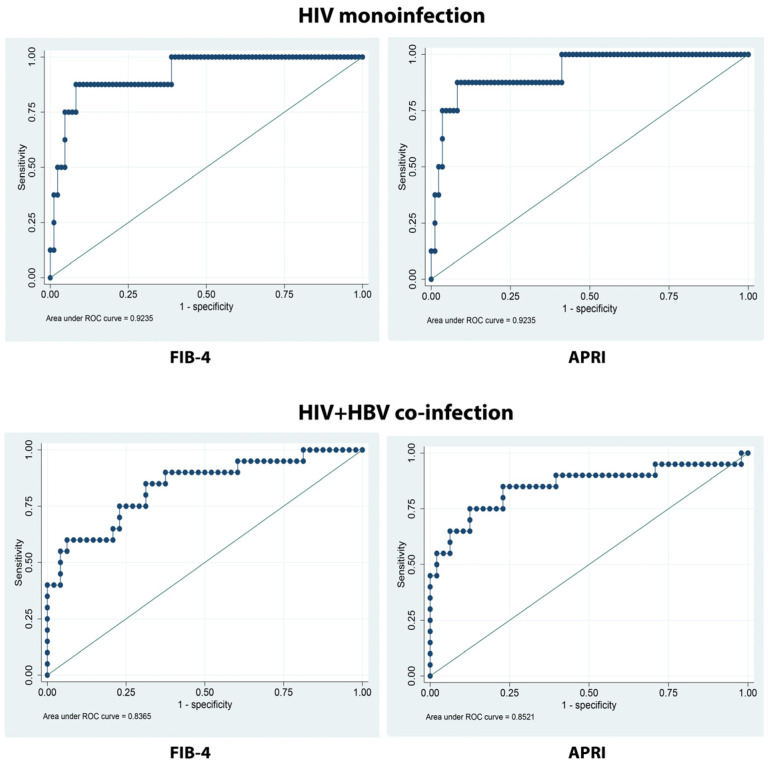
ROC curves for comparison between FIB-4 (results expressed in points) and APRI (results expressed in points) liver fibrosis scores and transient elastography (FibroScan^®^ results measured in kilopascals, kPa) in people living with HIV monoinfected and HIV-HBV co-infected.

In the two patient groups, both FIB-4 and APRI scores had similar specificities and sensitivities and positive and negative predictive values (Table 7). The APRI score showed slightly better performance based on sensitivity (37.84% vs. 37.50%/65.00% vs. 66.00%). The ROC curve for FIB-4 had an area under the curve (AUC) of 0.8271, whereas that for the APRI score was 0.8284 (Figure 8).

## 4. Discussion

### 4.1. Comparative Analysis between HIV Mono-Infected and HIV+HBV Co-infected Groups

Demographic variables. Both cohorts had balanced sex distribution (*p* = 0.645), suggesting sex may not significantly impact outcomes and clinical parameters in this study. However, further research is needed to fully understand sex dynamics, given its potential influence on disease progression and treatment outcomes [25,26]. A significant age difference was noted (*p* < 0.001), with the co-infected group being younger (median age 33 vs. 35 years), which could affect the disease course and treatment response. Younger age in the co-infected group might also indicate recent transmission events in the context of lower immunity [27], particularly for Hepatitis B [28], necessitating further epidemiological investigation.

Modes of transmission. Modes of transmission differed significantly between the groups (*p* < 0.001). The mono-infected group had a higher proportion of sexually transmitted cases, while the co-infected group showed more parenteral transmission, suggesting a higher prevalence among intravenous drug users or those exposed to blood products [29]. This indicates the need for targeted public health interventions.

Clinical parameters. There was no significant difference in AIDS stages between the groups (*p* = 0.814), indicating a similar rate of progression to AIDS with consistent HIV management across both groups.

Treatment duration. Treatment duration differed significantly (*p* < 0.001), with the co-infected group having a longer median treatment duration (22.5 years vs. 11 years) due to more complicated regimens and liver complications associated with Hepatitis B.

Virological and immunological parameters. No significant difference was found in viral suppression rates (*p* = 0.335), indicating that antiretroviral treatment is equally effective in both groups [30].

Metabolic and other health indicators. No significant difference in body weight status was observed (*p* = 0.918), suggesting similar nutritional status across both groups [31].

Liver fibrosis. A significant difference in FibroScan^®^ fibrosis stages was observed (*p* = 0.0028), with the co-infected group having more advanced fibrosis stages (F2, F3, F4). The higher prevalence of liver fibrosis in co-infected patients (29.41% vs. 8.60%) may be linked to the longer duration of antiviral treatment [32]. FIB-4 scores were halved in the co-infected group despite fewer patients with mild fibrosis due to younger age at diagnosis.

The FIB-4 score has inherent limitations that should be considered. The FIB-4 index is a useful tool for assessing liver fibrosis; however, it has some limitations. It was developed in a population that did not include extreme age groups (young or very old patients), so it may not perform as well in those groups. Additionally, the inclusion of age in the calculation makes it less reliable to use over time. Furthermore, the test may overestimate fibrosis in individuals with alcohol use owing to the presence of AST in the numerator. Finally, the cutoff values for HCV were different from those for NASH or HBV [23].

APRI scores did not differ significantly between groups. This is an expected but critical finding as hepatitis B infection is a known risk factor for liver fibrosis and cirrhosis [33,34]). These findings suggest that co-infected individuals require more intensive liver disease management and monitoring, emphasizing the need for integrated liver disease management in HIV treatment protocols.

Significant differences in transmission modes, age, treatment duration, and liver fibrosis stages were found between HIV-monoinfected and HIV+HBV co-infected groups. These differences underscore the need for tailored treatment management and public health interventions. Further research is required to confirm these findings and explore the complexities of managing co-infections.

### 4.2. Analysis of Risk Factors Associated with Liver Fibrosis

Patient sex. Male patients, especially those co-infected with HBV, may have higher liver fibrosis than females. Elevated FIB-4 and APRI scores in men suggest differences in liver disease progression due to factors like enzyme activity and comorbidities. The APRI score, significant in both groups, indicates men are at higher risk of fibrosis regardless of co-infection status. We must take into account that, in general, liver fibrosis is more prevalent in males than in females [35].

Current HIV viral load. No significant differences in fibrosis scores were observed based on viral load in both groups, suggesting viral load may not be a primary fibrosis driver. However, a detectable HIV viral load was near-significantly associated with higher FIB-4 scores, implying a link between uncontrolled HIV and liver disease progression.

Current CD4+ cell count. Patients with lower CD4+ counts (<200 cells/mL) showed more severe liver fibrosis, highlighting the role of immune status in liver health. Co-infected patients with low CD4 counts experienced rapid liver disease progression, likely due to heightened immunosuppression [36]. Low CD4 counts may independently indicate liver dysfunction even without HIV [37,38].

Clinical and immunological staging. In HIV patients, liver stiffness increases with advanced AIDS stages, peaking at stage C3. For HIV+HBV patients, higher elastography scores were seen across all stages, suggesting HBV co-infection exacerbates liver disease [39,40]. The highest scores in stage A2 suggest a nonlinear relationship between liver stiffness and AIDS stages in co-infected patients.

ESR. Elevated ESR levels correlated with higher FibroScan^®^ scores, indicating chronic inflammation’s role in liver fibrosis in HIV patients [41]. This discrepancy between fibrosis markers suggests that while ESR is a valuable inflammatory marker, it may not always be a reliable indicator of liver fibrosis in cases with multiple contributing factors, such as HBV co-infection.

Liver fibrosis. The similarity in FIB-4 and APRI scores across both groups suggests these markers may not effectively detect liver inflammation as FibroScan^®^ does. HBV co-infection may complicate the relationship between inflammation and fibrosis. FIB-4 and APRI scores are non-invasive markers for liver fibrosis in patients living with HIV and have been found to be significantly elevated in men. This may reflect differences in the progression of liver disease between the sexes, which could be attributed to factors such as disparities in hepatic enzyme activity, fat distribution, or the presence of comorbidities such as alcohol use [35]. In patients co-infected with hepatitis B, the hepatotropic virus could contribute to a more aggressive progression of liver disease in men [42].

The APRI score, which was significant in both groups, reinforces the notion that men in this cohort are at an increased risk of liver fibrosis irrespective of co-infection status.

HIV infection activates the immune system through constant viral replication and bacterial translocation, which, although weakened, is strong enough to sustain liver damage that finally leads to fibrosis [40,43].

### 4.3. Univariate Analysis

Patient age. In patients living with HIV, advancing age correlates with worsening liver fibrosis, likely due to prolonged exposure to risk factors such as HIV, alcohol, co-infections, and drug toxicity [32,35]. Conversely, in HIV+HBV co-infected patients, the impact of HBV, HIV interaction, and additional factors like alcohol use appear to overshadow the influence of age on liver fibrosis progression.

BMI. For HIV patients, BMI showed no correlation with liver fibrosis, suggesting that HIV-related factors might overshadow the influence of body mass. However, in HIV+HBV co-infected patients, a higher BMI moderately correlates with increased liver fibrosis, possibly due to fatty liver disease exacerbating fibrosis progression in the context of co-infection [31].

AST. AST levels strongly correlate with liver fibrosis scores in HIV patients, validating its use as an indicator of liver health influenced by antiretroviral therapy. This finding underscores the utility of AST as a contributing factor in liver fibrosis evaluation, particularly in the context of HIV infection [44]. We must consider the effect of antiretroviral therapy on the liver, which increases AST levels [45]. For co-infected patients, AST levels had similar trends.

Platelet count. Both groups showed significant negative correlations between platelet count and liver fibrosis scores, indicating that thrombocytopenia is a consistent marker of advanced liver disease across both patient populations, reflecting common mechanisms such as splenic sequestration and reduced thrombopoietin production in the liver [46].

ESR. In HIV patients, a strong positive correlation between ESR and liver stiffness suggests that ESR as a surrogate marker for fibrosis [41]. This was not specifically addressed in the co-infected group, but systemic inflammation likely plays a similar role.

CD4+ cells count. For HIV+HBV co-infected patients, a moderate correlation between CD4+ count and liver stiffness indicates that lower CD4+ levels are associated with greater liver fibrosis. This highlights the importance of managing immune status alongside viral infections to mitigate liver fibrosis [38,47], suggesting that antiretroviral therapy improving CD4+ counts could benefit liver health.

These findings could guide clinicians in monitoring liver health in patients living with HIV and in co-infected patients. They also highlighted the need for a more nuanced approach when managing co-infected patients, considering the broad range of variables that appear to influence liver health in this group.

### 4.4. Multivariate Analysis

Multivariate analyses provided a more nuanced understanding of the complex interplay between various factors influencing liver fibrosis in these patient groups.

In patients with HIV, the addition of HBV co-infection significantly increases the risk of liver fibrosis. Several mechanisms have been proposed to explain the increase in liver fibrosis.

HIV and HBV can interact at the molecular level, leading to increased viral replication and aggressive liver inflammation and damage. HIV can impair the immune system, making it more difficult to respond to HBV infection and potentially increasing the risk of chronicity. Both viruses can directly affect liver cells, with HIV enhancing fibrogenesis and HBV leading to cell death and regeneration in chronic infections [39]. Some antiretroviral drugs used to treat HIV have hepatotoxic potential and may cause more damage to the liver in the presence of HBV [39,40]. Additionally, when patients initiate ART to treat HIV, they may experience an inflammatory response as the immune system recovers (immune reconstitution inflammatory syndrome), which can worsen liver inflammation in the presence of underlying HBV infection.

Serum lipids and BMI. Serum lipid levels are inversely correlated with liver fibrosis. This suggests that elevated lipid levels may have a protective effect against liver fibrosis in people living with HIV. Conversely, a positive correlation was observed between BMI and liver fibrosis, indicating that an increasing BMI leads to a higher risk of liver fibrosis. This discrepancy can be attributed to metabolic factors associated with high BMI and may contribute to the development of liver fibrosis. An elevated BMI is often associated with increased systemic inflammation, which can contribute to liver injury and fibrosis. Conversely, specific lipid profiles may have anti-inflammatory or other protective effects on the liver. Additionally, antiretroviral therapies and HIV can influence metabolic profiles, potentially impacting both lipid levels and BMI differently [31].

AST and ALT levels, which were strongly correlated in the univariate analysis, lost their significance in the multivariate models, suggesting confounding effects. This is especially true for AST, which is a component of FIB-4 and APRI scores. Liver enzyme levels are associated with liver damage, which in turn is related to fibrosis [44,45].

ESR was consistently correlated in both cohorts, making it a critical parameter for liver health evaluation [41].

The contrasting results between the mono-infected and co-infected groups, particularly concerning sex and inflammatory markers, highlight the multifactorial etiology of liver fibrosis in these populations.

### 4.5. Comparison between Fibrosis Scores

The significant correlation between FIB-4 and APRI (rho values of 0.7905 and 0.7595 in both groups and 0.7696 in the total patient dataset with *p* < 0.001) suggests that these two non-invasive tests are closely related in their assessment of liver fibrosis, and they may be used interchangeably to some extent. However, it should be noted that while these two tests correlate well with each other, they had a weaker correlation with FibroScan^®^, which is often considered a more direct measure of liver stiffness.

The significant correlation observed between the APRI score and FibroScan^®^ only in the entire patient dataset (with a correlation coefficient of 0.2079 and *p*-value of 0.008) implies that the APRI score may have some utility in a broad clinical context. However, its utility in subgroups or individualized patient assessments may be limited. A 2019 study using an M probe for TE found a correlation coefficient of 0.32, which is slightly higher than ours [48].

In the evaluation of non-invasive FIB-4 and APRI scores compared to transient elastography, both scores demonstrated a high specificity of approximately 96%. This indicates that they possess an excellent ability to correctly identify patients without advanced fibrosis. However, their sensitivity was lower at approximately 30%, suggesting that they may not be effective in detecting most cases of liver fibrosis. However, a study from 2022 on HIV and HBV-co-infected patients demonstrated a sensitivity of 85.7% for FIB-4 > 1.5 and 76.0% for APRI > 0.5 [46]. The accuracy of FIB-4 and APRI can be affected by factors other than fibrosis, such as acute liver inflammation, extrahepatic cholestasis, and hemolysis [49].

The area under the ROC curve (AUC) for FIB-4 and APRI was similar, with values of 0.8271 and 0.8284, respectively. This suggests that both tests exhibit comparable overall performance in terms of differentiating between patients with and without liver fibrosis, although neither test is entirely flawless. These values are better than those in the original study on the development of the FIB-4 score [23]. A study in 2020 obtained AUCs of 0.697 and 0.698 for FIB-4 and APRI scores, respectively, which were lower than our AUCs [48].

Overall, although the APRI and FIB-4 scores were highly correlated and had high specificity, their sensitivity was relatively low. This suggests that they may be useful in ruling out advanced fibrosis but not in making a definitive diagnosis. Given these findings, a multimodal approach that incorporates these scores alongside FibroScan^®^ may provide a more comprehensive assessment of liver fibrosis in patients living with HIV and HIV+HBV co-infected patients.

## 5. Limitations

We encountered several limitations in this study: First, we did not have the possibility to check the activity of HBV infection, so we relied on the fact that the co-infected group was on stable antiretroviral therapy with antiretrovirals that are also active against HBV, such as Tenofovir, Lamivudine or Emtricitabine. Another limitation is the inability to assess alcohol consumption. HAART is known to influence serum levels of liver enzymes used for APRI and FIB-4 calculations. Drug-induced elevation of ALT and AST may yield false values and incorrectly infer liver fibrosis. To address this limitation, we used liver transient elastography to measure liver fibrosis in addition to APRI and FIB-4 scores.

The number of patients included in the study was rather small, as our hospital covers only the South-East region of Romania. Moving forward, we plan to expand this type of study to other universities in Romania to create a larger and more diverse sample. Additionally, we aim to establish national and international registries for longitudinal data collection to gather long-term information on HIV-related liver fibrosis.

## 6. Conclusions

In this study, we conducted an exhaustive evaluation of liver fibrosis markers, specifically focusing on FibroScan^®^, FIB-4, and APRI scores, across two patient cohorts: patients living with HIV and those co-infected with HIV and Hepatitis B. Our findings highlight several important considerations for clinicians and researchers.

While some markers showed potential correlations with liver fibrosis in univariate analysis, these associations were not consistent in multivariate analysis. This underscores the complexity of determining the factors influencing liver fibrosis in people living with HIV and the importance of considering multiple variables simultaneously.

Therefore, the combination of these laboratory markers can provide a meaningful, non-invasive assessment of liver fibrosis in this patient population. These results should encourage clinicians to consider these readily available tests as part of their routine evaluation of liver health in people living with HIV, particularly in settings where more advanced diagnostic options such as liver biopsies are not feasible.

Our study provides a nuanced understanding of the applicability and limitations of FibroScan^®^, FIB-4, and APRI as non-invasive markers for liver fibrosis in patients living with HIV and HIV plus Hepatitis B co-infected patients. Although these tests offer valuable benefits, particularly in terms of their high specificity, their low sensitivity necessitates a more comprehensive diagnostic approach for effective patient management.

Given the limitations in the sensitivity of FIB-4 and APRI, future research could focus on the development of new non-invasive markers or algorithms that offer better sensitivity without compromising specificity. Furthermore, longitudinal studies can provide insights into dynamic changes in these markers over time and their prognostic value.

## Figures and Tables

**Table 1:** Characteristics of patients living with HIV mono-infection (*n* = 93) vs. patients co-infected with HIV+HBV (n = 68). The significance levels for tests comparing the two patient groups were denoted as follows: * *p* < 0.05, ** *p* < 0.01, and *** *p* < 0.001.

Clinical Parameters		Paraclinical Parameters	
Parameter	Median(IQR) No. (%) (HIV Only/HIV+HBV)	Parameter	Median(IQR) No. (%) (HIV Only/HIV+HBV)
		Laboratory findings	
Age at diagnosis ***	24 (16–37)/11.5 (7–22.5)	CD4 count at diagnosis, cells/mm^3^	251 (89–456)/266.5 (113.5–452)
Age (current) **	35 (33–44)/33 (32–34)	CD4 count (current), cells/mm^3^	401 (238–584)/445 (198.5–584.5)
Sex		CD8, cells/mm^3^	595 (387–865)/645 (415–877)
Women	47 (50.54)/31 (45.59)	CD4/CD8	0.64 (0.4–0.98)/0.68 (0.36–1.03)
Men	46 (49.46)/37 (54.41)	ESR, mm/1 h	20 (11–40)/22.5 (10–42.5)
Overweight		HIV viral load at diagnosis (c/mL) *	26.400 (918–175,000)/7517.5 (336–75,362)
Normal weight	58 (62.37)/41 (60.29)	HIV viral load (current) > 40 c/mL	
Overweight	35 (37.63)/27 (39.71)	<40 c/mL	62 (67.39)/50 (75.76)
HIV transmission route ***		>40 c/mL	30 (32.61)/16 (24.24)
Sexual	65 (70.65)/20 (29.41)	AST	28 (23–39)/33 (24–45.5)
Parenteral	26 (28.26)/48 (70.59)	ALT	27 (19–40)/33.5 (19.8–52)
Vertical	1 (1.09)/0 (0)	TROMB	225 (173–264)/226 (173.5–273.5)
Treatment duration *** (years)	11 (5–19)/22.5 (13–26)	CRE	0.83 (0.72–0.96)/0.82 (0.74–0.97)
HIV infection stage		EGFR	88.18 (76.7–101.12)/88.235 (77.28–103.11)
A1	3 (3.23)/1 (1.47)	UREA	28 (22–36)/25 (21–29)
A2	6 (6.45)/2 (2.94)	BMI	23.81 (20.89–27.74)/23.66 (20.8–25.93)
B1	2 (2.15)/3 (4.41)	Liver fibrosis assessment	
B2	19 (20.43)/12 (17.65)	FibroScan^®^ (kPa) **	4 (4.9–5.8)/5.75 (4.4–7.7)
B3	21 (22.58)/21 (30.88)	FibroScan^®^ stage	
C1	2 (2.15)/1 (1.47)	F0–F1 ***	85 (91.4)/48 (70.59)
C2	6 (6.45)/3 (4.41)	F2	5 (5.38)/8 (11.76)
C3	34 (36.56)/25 (36.76)	F3 **	1 (1.08)/9 (13.24)
		F4	2 (2.15)/3 (4.41)
		FIB-4 at diagnosis **	0.69 (0.36–1.14)/0.38 (0.16–0.77)
		FIB-4 (current)	0.9 (0.68–1.54)/ 0.84 (0.65–1.34)
		APRI at diagnosis	0.36 (0.22–0.83)/ 0.42 (0.22–0.74)
		APRI (current)	0.34 (0.23–0.57)/ 0.36 (0.23–0.53)

IQR: Interquartile range.

**Table 2:** Univariate correlations between risk factors and liver fibrosis in patients living with HIV mono-infection and HIV+HBV co-infection.

Parameter	FibroScan^®^			FIB-4			APRI		
	Total	HIV Only	HIV+ HBV	Total	HIV Only	HIV+ HBV	Total	HIV Only	HIV+ HBV
Sex male	0.101	0.003	0.220 *	0.118	0.067	0.167	0.158 *	0.123	0.191
Age at diagnosis	−0.081	0.039	−0.138	0.077	0.230 *	−0.045	−0.003	0.153	−0.125
BMI	0.093	0.040	0.267 *	−0.020	−0.006	−0.041	−0.050	−0.061	−0.029
AST	0.273 ***	0.353 **	0.142	0.563 ***	0.400 ***	0.815 ***	0.861 ***	0.901	0.903 ***
ALT	0.071	0.048	0.244	0.067	0.091	0.077	0.477 ***	0.710	0.239 *
Platelet count	−0.149 *	−0.192	−0.113	−0.531 ***	−0.582 ***	−0.520 ***	−0.398 ***	−0.331 **	−0.492 ***
HIV viral load	0.038	0.076	0.014	0.045	0.053	0.059	0.046	0.034	0.107
HIV viral load at diagnosis	−0.045	−0.036	−0.023	−0.049	−0.057	−0.044	−0.076	−0.060	−0.096
CD4^+^ count	0.003	−0.152	0.263*	−0.158 *	−0.217 *	−0.114	0.002	0.136	−0.124
CD4^+^ count at diagnosis	0.022	−0.080	0.145	−0.090	−0.124	−0.064	−0.118	−0.163	−0.074
ESR	0.178 *	0.398 ***	−0.087	0.016	0.123	−0.063	0.032	0.153	−0.096
Time passed since diagnosis	0.102	−0.001	0.127	0.019	−0.061	0.086	−0.042	−0.223 *	0.084
Overweight	0.152 *	0.076	0.252 *	−0.036	−0.060	−0.018	−0.031	−0.095	0.025

The significance levels for tests comparing the two patient groups were denoted as follows: * *p* < 0.05, ** *p* < 0.01, and *** *p* < 0.001.

**Table 3:** Multivariate analysis of parameters correlated with the risk of developing advanced fibrosis estimated using transient elastography (FibroScan^®^) in patients in both groups.

	Total		HIV Mono-Infected		HIV+HBV Co-Infected	
	Odds Ratio	*p*	Odds Ratio	*p*	Odds Ratio	*p*
CO-INFECTION HIV+HVB	5.726	0.003	-	-	-	-
MALE SEX	5.338	0.014 *	11.55799	0.182	7.674568	0.085
AGE AT DIAGNOSIS	0.988	0.792	0.8458012	0.379	1.011804	0.797
TOTAL LIPIDS	0.999	0.957	0.9602675 *	0.009	0.9997993	0.872
IMC	1.085	0.077	6.116016 *	0.006	1.558819	0.117
AST	1.010	0.132	1.738618 *	0.006	1.002563	0.747
ALT	0.996	0.231	0.8081814 *	0.008	1.013053	0.113
THROMBOCYTES	0.998	0.637	1.021767	0.212	0.9929535	0.488
VL HIV AT DIAGNOSIS	0.9999986	0.316	0.9999362 *	0.003	0.9999999	0.738
VL HIV (CURRENT)	1.000003	0.253	1.000048 *	0.002	1.000016 *	0.050
CD4 COUNT (CELLS/MM3) AT DIAGNOSIS	0.9994	0.596	0.9773227	0.005	1.000147	0.908
CD4 COUNT (CELLS/MM3)	1.0008	0.566	1.0068	0.327	1.001445	0.332
ESR (MM/1 H)	1.030 *	0.005	1.378 *	0.032	1.028 *	0.045
TIME SINCE DIAGNOSIS	1.042	0.545	0.986	0.964	1.049	0.629
CONSTANT	0.002	0.016	6.73 × 10^−21^	0.001	3.73 × 10^−7^	0.070

ESR: erythrocyte sedimentation rate. VL: viral load. The significance levels for tests comparing the two patient groups were denoted as follows: * *p* < 0.05.

**Table 4:** Multivariate analysis of parameters correlated with the risk of developing advanced fibrosis estimated using the FIB-4 score, in patients from both groups.

	Total		HIV Mono-Infected		HIV+HBV Co-Infected	
	Odds Ratio	*p*	Odds Ratio	*p*	Odds Ratio	*p*
CO-INFECTION HIV+HBV	0.81	0.665	-	-	-	-
MALE SEX	1.991	0.124	2.126	0.136	7.197	0.128
TOTAL LIPIDS	1.001 *	0.027	1.002 *	0.016	1.001 *	0.267
BMI	1.041	0.469	1.089 *	0.057	0.727	0.014
VL HIV AT DIAGNOSIS	1	0.860	0.9999998	0.750	1.000001	0.556
VL HIV (CURRENT)	1	0.795	1.000001	0.571	0.99999	0.750
CD4 COUNT (CELLS/MM3) AT DIAGNOSIS	1.0001	0.863	0.9991	0.360	1.0012	0.389
CD4 COUNT (CELLS/MM3)	0.9991	0.352	0.9996	0.781	0.9985	0.391
ESR (MM/1 H)	1.005	0.594	0.992	0.591	1.019	0.154
TIME SINCE DIAGNOSIS	0.961	0.143	0.975	0.469	0.938	0.228
CONSTANT	0.065	0.047	0.017	0.002	48.395	0.217

ESR: erythrocyte sedimentation rate. VL: viral load. The significance levels for tests comparing the two patient groups were denoted as follows: * *p* < 0.05.

**Table 5:** Multivariate analysis of parameters correlated with risk of developing advanced fibrosis estimated by APRI score in patients from both groups.

	Total		HIV Mono-Infected		HIV+HBV Co-Infected	
	Odds Ratio	*p*	Odds Ratio	*p*	Odds Ratio	*p*
CO-INFECTION HIV+VHB	0.710	0.467	-	-	-	-
MALE SEX	1.729	0.228	1.364	0.555	4.161	0.179
ALT (UI/L)	1.040 *	<0.001	1.044 *	0.014	1.039 *	0.009
TOTAL LIPIDS	1.001	0.118	1.003 *	0.039	0.874	0.590
BMI	0.922	0.124	0.957	0.557	1.00001	0.236
VL HIV AT DIAGNOSIS	1.000	0.737	0.9999	0.857	0.99999	0.255
VL HIV (CURRENT)	1.000	0.992	1.000	0.999	0.99991	0.983
CD4 COUNT (CELLS/MM3) AT DIAGNOSIS	0.99996	0.952	0.9998	0.857	0.9991	0.949
CD4 COUNT (CELLS/MM3)	0.99847 *	0.071	0.9973 *	0.063	0.9996	0.769
ESR (MM/1 H)	1.005	0.605	0.992	0.419	1.013	0.405
TIME SINCE DIAGNOSIS	1.008	0.739	0.998	0.979	1.025	0.505
CONSTANT	0.286	0.337	0.120	0.204	0.548	0.824

ESR: erythrocyte sedimentation rate. VL: viral load. The significance levels were denoted as follows: * *p* < 0.05.

**Table 6:** Correlation between paraclinical scoring systems used to evaluate liver fibrosis and transient elastography (FibroScan^®^).

Patient Group		FibroScan^®^ Rho ^¥^ (*p* Values)	FIB-4 Rho ^¥^ (*p* Values)	APRI Rho ^¥^ (*p* Values)
Patients HIV-monoinfected	FibroScan^®^	-		
	FIB-4	0.112 (0.253)	-	
	APRI	0.1320 (0.2073)	0.7905 (<0.001)	-
Patients co-infected HIV+HBV	FibroScan^®^	-		
	FIB-4	0.0835 (0.498)	-	
	APRI	0.2615 (0.313)	0.7595 (<0.001)	-
TOTAL	FibroScan^®^	-		
	FIB-4	0.0822 (0.300)	-	
	APRI	0.2079 (0.008)	0.7696 (<0.001)	-

^¥^ Spearman’s correlation coefficient.

**Table 7:** Sensitivity and specificity of FIB-4 and APRI scores for liver fibrosis compared with transient elastography (FibroScan^®^).

Liver Fibrosis Score	Sensitivity %	Specificity %	Positive Predictive Value	Negative Predictive Value
People living with HIV mono-infection				
FIB-4	37.50%	98.82%	75.00%	94.38%
APRI	37.84%	98.31%	75.20%	94.57%
HIV+HBV co-infected patients				
FIB-4	60.00%	91.67%	75.00%	84.62%
APRI	65.00%	91.67%	76.47%	86.27%

## Data Availability

The data presented in this study are available upon request from the corresponding author. The data are not publicly available due to the patient’s personal data protection policy of the University and Hospital.
